# The ABC-Stroke Score Refines Stroke Risk Stratification in Patients With Atrial Fibrillation at the Emergency Department

**DOI:** 10.3389/fmed.2022.830580

**Published:** 2022-06-27

**Authors:** Jan Niederdöckl, Julia Oppenauer, Sebastian Schnaubelt, Filippo Cacioppo, Nina Buchtele, Alexandra-Maria Warenits, Roberta Laggner, Nikola Schütz, Magdalena S. Bögl, Gerhard Ruzicka, Sophie Gupta, Martin Lutnik, Safoura Sheikh Rezaei, Michael Wolzt, Harald Herkner, Hans Domanovits, Anton N. Laggner, Michael Schwameis, Ziad Hijazi

**Affiliations:** ^1^Department of Emergency Medicine, Medical University of Vienna, Vienna, Austria; ^2^Department of Clinical Pharmacology, Medical University of Vienna, Vienna, Austria; ^3^Department of Medicine I, Medical University of Vienna, Vienna, Austria; ^4^Department of Orthopedics and Trauma-Surgery, Medical University of Vienna, Vienna, Austria; ^5^Department of Medical Sciences, Cardiology, Uppsala Clinical Research Center, Uppsala University, Uppsala, Sweden

**Keywords:** symptomatic atrial fibrillation, stroke, prediction score, performance evaluation, validation, biomarkers

## Abstract

**Aims:**

To evaluate the performance of the ABC (Age, Biomarkers, Clinical history) and CHA_2_DS_2_-VASc stroke scores under real-world conditions in an emergency setting.

**Methods and Results:**

The performance of the biomarker-based ABC-stroke score and the clinical variable-based CHA_2_DS_2_-VASc score for stroke risk assessment were prospectively evaluated in a consecutive series of 2,108 patients with acute symptomatic atrial fibrillation at a tertiary care emergency department. Performance was assessed according to methods for the development and validation of clinical prediction models by Steyerberg et al. and the Transparent Reporting of a Multivariable Prediction Model for Individual Prognosis or Diagnosis. During a cumulative observation period of 3,686 person-years, the stroke incidence rate was 1.66 per 100 person-years. Overall, the ABC-stroke and CHA_2_DS_2_-VASc scores revealed respective c-indices of 0.64 and 0.55 for stroke prediction. Risk-class hazard ratios comparing moderate to low and high to low were 3.51 and 2.56 for the ABC-stroke score and 1.10 and 1.62 for the CHA_2_DS_2_-VASc score. The ABC-stroke score also provided improved risk stratification in patients with moderate stroke risk according to the CHA_2_DS_2_-VASc score, who lack clear recommendations regarding anticoagulation therapy (HR: 4.35, *P* = 0.001). Decision curve analysis indicated a superior net clinical benefit of using the ABC-stroke score.

**Conclusion:**

In a large, real-world cohort of patients with acute atrial fibrillation in the emergency department, the ABC-stroke score was superior to the guideline-recommended CHA_2_DS_2_-VASc score at predicting stroke risk and refined risk stratification of patients labeled moderate risk by the CHA_2_DS_2_-VASc score, potentially easing treatment decision-making.

## Introduction

The more than 13 million strokes that occur worldwide each year drive constant efforts to optimize patient management (1, 2). Up to one-third of incident ischemic strokes are cardio-thromboembolic in source and associated with atrial fibrillation (AF) (2, 3). Adequate long-term management of AF needs to be initiated early, preferably during the patient‘s initial presentation to the emergency department (2, 4).

Risk stratification schemes for AF patients have been continuously improving over recent years (5–7). Routine clinical risk assessment is widely based on the CHA_2_DS_2_-VASc score. While the CHA_2_DS_2_-VASc score uses clinical variables, the more-recent ABC (Age, Biomarkers, Clinical history)-stroke score incorporates age and clinical history of previous stroke/transient ischemic attack with two cardiac biomarkers: high sensitivity cardiac troponin T (hs-cTnT) and the pro-B-type natriuretic peptide N-terminal fragment (NT-proBNP) (8–11).

Risk prediction based on the ABC-stroke score appears superior to that obtained with the CHA_2_DS_2_-VASc score (6, 12). However, clinical presentation and medical history of patients with AF can vary greatly, which strongly impacts relevant biomarkers. Therefore, a detailed characterization of score performance at the subgroup level is essential. In the emergency setting, no optimal score for stroke prediction in patients with AF has yet been defined (13, 14). The current study evaluated the performance of the biomarker-based ABC-stroke score and the clinical variable-based CHA_2_DS_2_-VASc score, which is recommended by current guidelines, in patients with acute symptomatic AF in the emergency department.

## Methods

### Ethical Approval

This study, which was conducted in accordance with ICH-GCP guidelines and the Declaration of Helsinki, was approved by the Ethics Committee of the Medical University of Vienna (1568/2014); Patients gave written consent before participation. The study was registered at clinicaltrials.gov (NCT03272620).

### Emergency Department Cohort

This study included a consecutive series of patients with acute, symptomatic, hemodynamically stable, first-detected, or recurrent nonpermanent AF who presented to the emergency department of the Medical University of Vienna, a tertiary care academic institution, from 2011 to 2018. Only patients with acute symptoms strongly suggestive of tachyarrhythmia, such as palpitations, whose 12-lead ECG confirmed the presence of AF were eligible. Data were prospectively collected within the framework of a local, standardized AF registry that was previously described (13). Patients with permanent AF (n = 139) and cases of hemodynamic instability in which AF occurred as an epiphenomenon of the underlying disease (n = 87) were not included in the registry. Prospective enrolment into the registry required that the patient's AF be confirmed by a 12-channel ECG and that they provided informed consent. Demographic data, comorbidities, medication intake, previous electrical cardioversion attempts, CHA_2_DS_2_-VASc score, blood gas analysis, laboratory results, vital signs (heart rate, blood pressure, and oxygen saturation), symptoms and time of onset, and previous treatments were recorded. Treatment strategies for acute control of rate and rhythm were based on the European Society of Cardiology guidelines and considered the patient's left ventricular function, hemodynamics, medication intake (such as anticoagulants), and comorbidities (2, 4).

### Laboratory Values and Measurements

Serum NT-proBNP and hs-cTnT assays were processed on a Cobas E602 Module Console (ECLIA, Roche Diagnostics GmbH, Mannheim, Germany) with coefficients of variation and reference ranges of 5.7% and 0–14 ng/L for hs-cTnT and 3.7% and 0–125 pg/mL for NT-proBNP. The limit of blank was 3 ng/L for hs-cTnT. The limits of detection were 5 ng/L for hs-cTnT and 5 pg/mL for NT-proBNP (according to CLSI EP17-A guidelines) (13).

### Cohorts Used to Derive the ABC-Stroke and CHA_2_DS_2_-VASc Scores

A comprehensive report on the development of the ABC-stroke score based on the ARISTOTLE trial has recently been published (8). Briefly, 18,201 patients with AF at risk of stroke were randomized to receive oral anticoagulation with either warfarin or apixaban. The 14,701 patients with available biomarkers for ABC-stroke score derivation were followed for a median time of 1.9 years. The development, validation, and performance of the CHA_2_DS_2_-VASc score has been described in detail previously (5).

### Outcome

The outcome was defined following the International Classification of Diseases (ICD), 10th Revision, German Modification (2020) and assessed in a total of 2,108 patients that were not censured for treatment or medical history. The outcome was ischemic stroke (I63.-) and included patients with haemorrhagic transformation (I.69.3) but not primary intracerebral hemorrhage (I61.-). In addition, trained study fellows used a structured telephone interview with the patient or, in cases of death or communication difficulties, the next of kin to complete a standardized follow-up questionnaire developed at our institution to systematically assess the outcome. The observation period ended on either the last date of follow up or the date of death.

### Statistical Methods

We present categorized data as absolute counts and relative frequency, and continuous data as median and 25–75% interquartile range (IQR). The 1-year risk of stroke was calculated for each patient using both the ABC-stroke score and the CHA_2_DS_2_-VASc score, irrespective of stroke history or whether or not the patient was on anticoagulation therapy. The discriminatory capabilities of the scores were evaluated using Harrel's c-indices (15), Kaplan-Meier curves, and hazard ratios. These were applied to predefined risk classes with 0–1%, 1–2%, or >2% risk of stroke within 1 year. The predictive capabilities of the ABC-stroke score and CHA_2_DS_2_-VASc risk score were then compared (5, 6, 16). C-indices were compared utilizing 1,000-fold bootstraps. We performed sensitivity analyses taking into account anticoagulation status, history of ischemic stroke, the presence of acute heart failure and acute coronary syndrome. The net clinical benefits of the ABC-stroke score and the CHA_2_DS_2_-VASc score were determined with a decision curve analysis (17). In this context, net clinical benefit was defined as the relationship between the benefit of treating those who need treatment and the harm of treating those who do not need treatment. Decision curve analysis allows the evaluation of net clinical benefit of a prognostic tool over a range of threshold probabilities of having a positive outcome. Net clinical benefit is calculated as $\frac{true\;positives}{n}\;-\;\frac{false\;positives}{n}\;*\left(\frac{pt}{1-\;pt}\right)$, where *n* is the total number of patients, and *pt* is the threshold probability of having a positive outcome. True and false positives are calculated using *pt* as the cut-off point for determining a positive or negative result. By calculating and plotting the net benefit for all reasonable thresholds, the decision curve is then determined, allowing comparison of several predictive models. The model with the highest net benefit for a given threshold is assumed to be the preferred one. This is shown in a coordinate system with possible thresholds for stroke risk on the x-axis and the net benefit per patient on the y-axis. In addition, the predictive capabilities of the ABC-stroke score, discriminatory power, and net clinical benefit, were assessed across CHA_2_DS_2_-VASc risk classes. The analyses were conducted in accordance with the recommendations on the derivation and validation of prediction models proposed by Steyerberg et al. (1). Reporting followed the Transparent Reporting of Multivariable Prediction Model for Individual Prognosis or Diagnosis (TRIPOD) statement (18). Missing data were included as separate categories for each variable as appropriate. Stata/BE 17.0 for Mac (StataCorp, College Station, TX 77845, USA) was used for data analysis. Generally, a two-sided *P*-value < 0.05 was considered to be statistically significant.

## Results

### Demographics and Patient Characteristics

A consecutive series of 2,108 patients with acute symptomatic AF were included in the study. Patient characteristics are shown in Table 1. The median age was 68 years (IQR 59–76), 911 patients (43%) were female. The median time from onset of arrhythmia-related symptoms to presentation was 6 h (IQR 2–20). Of the 2,108 included patients, 733 patients (34.7%) were on anticoagulation therapy at presentation, and 1,344 (63.8%) were on anticoagulants at the time of discharge or transfer from the emergency department (Supplementary Table 1). Within this cohort, 123 patients (5.8%) had a history of ischemic stroke.

**Table 1:** Demographics and baseline characteristics of study patients by CHA_2_DS_2_-VASc risk classes.

	**Total**		**Treatment recommendation based on CHA**_**2**_ **DS**_**2**_ **-VASc**					
			**No**		**To be considered** **non-sex CHA**_**2**_**DS**_**2**_**-VASc**		**Yes**	
			**<1**		**1**		**>1**	
	***n* = 2,108**	Available^*^(*n*)	***n* = 219**	Available^*^(*n*)	***n* = 437**	Available^*^(*n*)	***n* = 1,452**	Available^*^(*n*)
**General characteristics**
Age, years (IQR)	68 (59–76)		51 (37–58)		62 (53–68)		73 (66–80)	
Female sex, *n* (%)	911 (43)		87 (40)		163 (37)		661 (46)	
**Comorbidities**
Heart failure, *n* (%)	369 (17.5)		4 (1.8)		59 (13.5)		306 (21.1)	
Hypertension, *n* (%)	1,236 (58.6)		0 (0.0)		200 (45.8)		1,036 (71.3)	
Diabetes mellitus, *n* (%)	344 (16.3)		0 (0.0)		12 (2.7)		332 (22.9)	
Prior stroke, *n* (%)	123 (5.8)		0 (0.0)		0 (0.0)		123 (8.5)	
Coronary artery disease, *n* (%)	383 (18.2)		0 (0.0)		26 (5.9)		357 (24.6)	
Prior myocardial infarction, *n* (%)	195 (9.3)		0 (0.0)		7 (1.6)		188 (12.9)	
Peripheral artery disease, *n* (%)	93 (4.4)		0 (0.0)		3 (0.7)		90 (6.2)	
**AF history**
First AF episode, *n* (%)	29 (1.4)		1 (0.0)		8 (0.4)		20 (0.9)	
Heart rate, bpm (IQR)	131 (110–150)	1,097	135 (108–159)	148	134 (119–151)	208	129 (108–147)	714
Duration of AF symptoms, h (IQR)	6 (2–20)	846	4 (1–10)	121	6 (2–18)	190	7 (3–24)	535
**Laboratory**
NT-proBNP, pg/ml (IQR)	636 (150–2,153)	2,108	244 (64–782)	219	465 (133–1,588)	437	932 (192–2,433)	1,452
hs-Troponin T, ng/l (IQR)	12 (5–23)	2,108	7 (3–13)	219	8 (4–16)	437	13 (6–28)	1,452
**Stroke risk scores**
CHA_2_DS_2_-VASc (IQR)	3 (1–4)	1,984	0 (0–0)	219	1 (1–2)	437	3 (3–4)	1,328
ABC-stroke risk, 1-year (IQR)	0.9 (0.5–1.5)	2,018	0.5 (0.3–0.8)	219	0.7 (0.5–1.2)	437	1.1 (0.6–1.7)	1,452
**Outcome**
Stroke, *n* (%)	61 (3.0)		1 (1.4)		14 (4.7)		46 (3.2)	
Time to event, months (IQR)	12.1 (3.5–32.2)		22.1 (1.0–33.8)		14.2 (3.9–24.4)		12.4 (2.8–32.2)	

*AF, atrial fibrillation; hs-Troponin T, high-sensitivity Troponin T; NT-proBNP, N-terminal pro-B-type natriuretic peptide*.

* *The number of patients for whom the variable was available*.

### Outcome

Ischemic stroke occurred in 61 patients during the median follow-up period of 23 months (IQR: 12–39). Incidence rates of strokes per 100 person-years according to ABC-stroke score and CHA_2_DS_2_-VASc score risk classes are presented in Table 2 and corresponded to an overall incidence rate of 1.66 per 100 person-years.

**Table 2:** Stroke incidence rates and hazard ratios for the ABC and CHA_2_DS_2_-VASc stroke risk classes.

**Incidence rates and hazard ratios**	***N***	**Events**	**Incidence rate^°^**	**Hazard ratio**	***p***
**ABC-stroke score**					
Low risk (<1%)	1,135	16	0.79 (0.49–1.30)	1.00	Ref
Medium risk (1–2%)	731	37	2.87 (2.08–3.96)	3.51 (1.95–6.31)	<0.001
High risk (>2%)	242	8	2.12 (1.06–4.23)	2.56 (1.10–5.98)	<0.030
**CHA**_**2**_ **DS**_**2**_ **VASc**					
Low risk (<1%^*^)	504	10	1.21 (0.70–2.24)	1.00	Ref
Medium Risk (1–2%^**^)	409	10	1.29 (0.70–2.40)	1.10 (0.46–2.64)	<0.832
High risk (>2%^***^)	1,195	41	1.97 (1.45–2.68)	1.62 (0.81–3.24)	<0.170

* *CHA_2_DS_2_-VASc ≤ 1*.

** *CHA_2_DS_2_-VASc = 2*.

*** *CHA_2_DS_2_-VASc > 2 according to Oldgren et al. (6)*.

° *Per 100 person years*.

### Performance Evaluation

The hazard ratios for the ABC-stroke score and CHA_2_DS_2_-VASc score are presented in Table 2. Cumulative event rates and c-indices for both scores are shown in Table 3. Overall, the ABC-stroke score had a c-index for stroke prediction of 0.64, and the CHA_2_DS_2_-VASc score had a c-index of 0.55. In patients with moderate stroke risk according to the CHA_2_DS_2_-VASc score, the ABC-stroke score provided improved risk stratification (hazard ratio: 4.35, *P* = 0.001). Results from sensitivity analyses in important clinical subgroups are available in Supplementary Table 2. The ABC-stroke score performed consistently in important subgroups, such as the anticoagulation status at discharge, the presence of acute heart failure, and in patients without acute coronary syndromes.

**Table 3:** C-indices for the ABC-stroke and CHA_2_DS_2_-VASc scores.

**C-indices**	***N***	**Events**	**Harrell's C**
**ABC-stroke score**			
All patients with acute AF	2,108	61	0.64 (0.57–0.70)
Consider anticoagulation^*^	437	14	0.66 (0.51–0.80)
**CHA**_**2**_ **DS**_**2**_ **VASc**			
All patients with acute AF	2,108	61	0.55 (0.49–0.60)

*C-indices with 95% confidence intervals in parentheses for the ABC-stroke and CHA_2_DS_2_-VASc scores in the total cohort (n = 2,108) and in patients classified as “consider anticoagulation” according to ESC guidelines (n = 437) (2, 4)*.

* *Non-sex CHA_2_DS_2_-VASc = 1*.

### Decision Curve Analysis

**Figure 1:**
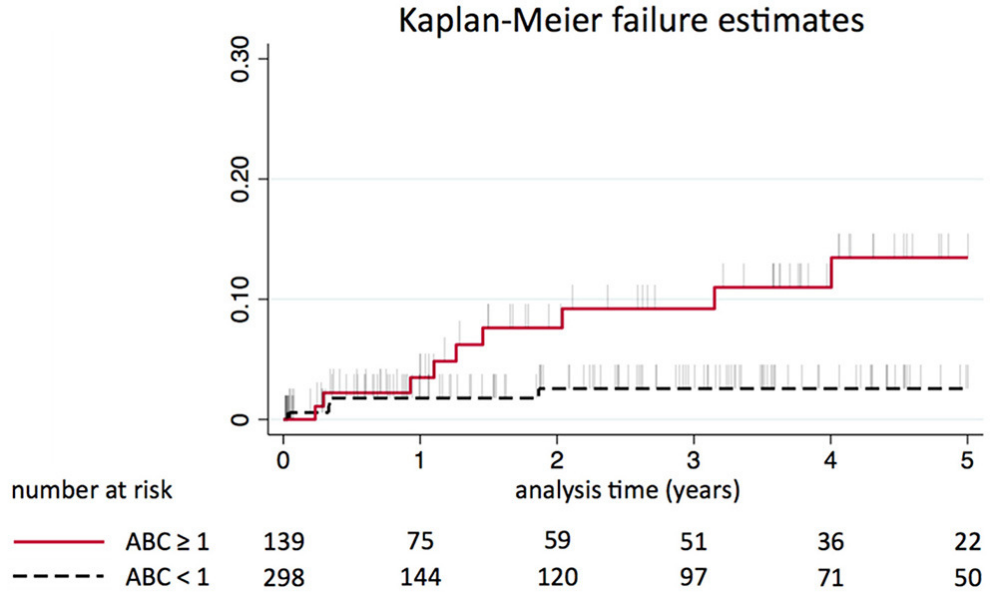
Kaplan-Meier estimated event rate for patients labeled as moderate risk by the CHA_2_DS_2_-VASc score stratified by ABC-stroke risk classes (≥1 vs. <1% risk of stroke per 1 year).

**Figure 2:**
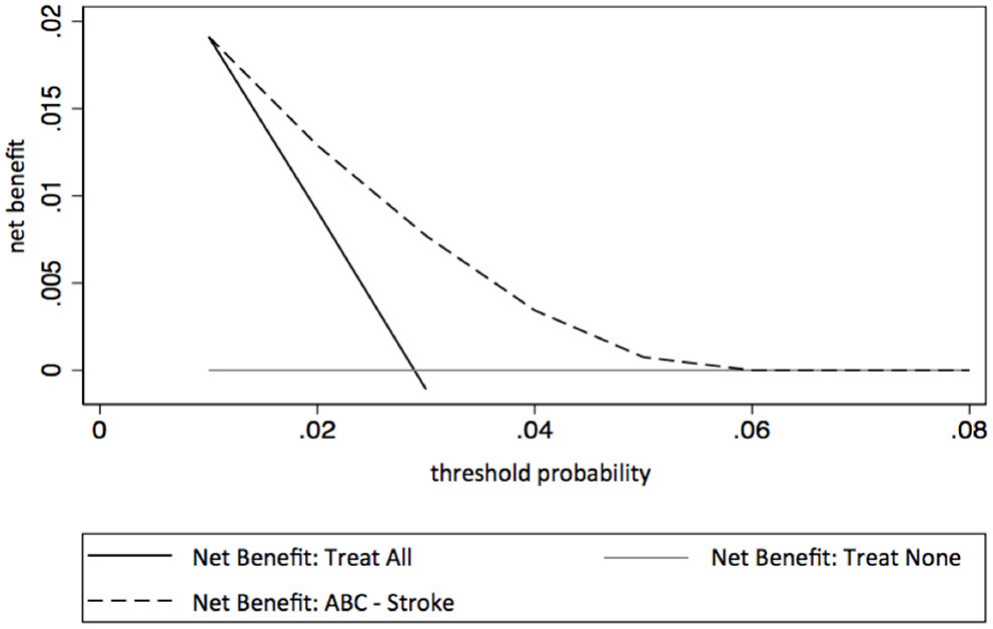
Decision curve analysis for the ABC-stroke score. Net benefit (y-axis) reflects whether basing ischemic stroke risk prediction on the ABC*-*stroke score provides greater benefit than harm. The unit of net benefit is true positives (ischemic strokes) per patients. A net benefit of 0.01 means that using the ABC*-*stroke score increases the number of correctly predicted ischemic strokes by 1 out of 100 target patients, without changing the number of false-positive stroke predictions. Threshold probability (x-axis) refers to the cut-offs of predicted ischemic stroke risk used to decide treatment (19, 20).

The decision curve analyses showed a net benefit for using either scoring system. The benefit of using the ABC-stroke score was superior to that of the CHA_2_DS_2_-VASc score over the 1–5% decision threshold range and was most prominent in patients labeled moderate risk by the CHA_2_DS_2_-VASc score, for whom anticoagulation may be considered (Figures 1, 2).

The Kaplan-Meier estimated event rate stratified by CHA_2_DS_2_-VASc risk classes are shown in the Supplementary Figure 1.

## Discussion

We evaluated the performance of the biomarker-based ABC-stroke score compared to the widely used clinical variables-based CHA_2_DS_2_-VASc score in a large series of patients admitted to the Medical University of Vienna Emergency Department with acute symptomatic AF. The ABC-stroke score predicted stroke better than the CHA_2_DS_2_-VASc score.

Because of their overall high stroke rates, it is important that patients with AF receive adequate anticoagulation treatment whenever indicated (2, 4, 21, 22). Thus, published guidelines recommend the use of prediction tools to guide appropriate treatment (2). While the clinical CHA_2_DS_2_-VASc score is recommended for daily practice, biomarker-driven scores are gaining in importance (2, 6, 8–10).

The ABC-stroke score has been validated, and its superiority over other prediction models has been shown in several patient cohorts (6, 8, 12, 23, 24). Its discriminative ability is based on four variables: age, the history of previous stroke or transient ischemic attack, and two cardiac biomarkers (hs-cTnT and NT-proBNP) (8). While the ubiquitous availability of the two cardiac biomarkers represents an advantage, their sensitivity to various modifying factors should be kept in mind (6, 13, 14). Both biomarkers included in the ABC-stroke score may be affected by myocardial oxygen demand, which is altered by heart rate and tachyarrhythmia severity (11, 14, 25). Since the ABC-stroke score was developed in clinically stable patients with AF treated with oral anticoagulation and enrolled in a randomized controlled trial, its usefulness in an unselected “real-world” cohort of patients presenting with acute AF to an emergency department has not yet been determined.

To our knowledge, the current study is the first to show that the ABC-stroke score performs well in a relatively large and diverse emergency cohort. This present study cohort had a wide spectrum of comorbidities and included patients with significantly altered vital parameters, such as ongoing tachyarrhythmia with median heart rates of 128 bpm at baseline (IQR: 110–131) (13, 26). Despite such conditions potentially influencing the cardiac biomarkers in the ABC-stroke score, it outperformed the guideline-recommended CHA_2_DS_2_-VASc score that is based solely on categorical variables (6). The wide inclusion criteria in the study also permitted clinically important subanalyses. The ABC-stroke score performed consistently in important subgroups, irrespective of anticoagulation status and heart failure. However, the ABC-stroke score performed less well in patients with concomitant acute coronary syndrome. However, this is expected because such a condition substantially alters cardiac troponin levels. Therefore, acute coronary syndrome should be ruled out before stroke risk stratification. The ABC-stroke score yielded a higher stroke risk in moderate-risk patients than high-risk patients. This was most likely due to a lower proportion of patients in the moderate-risk group taking oral anticoagulants (27). Nevertheless, decision curve analysis indicated superior treatment decision-making with the ABC-stroke score compared to the CHA_2_DS_2_-VASc score. Additionally, the ABC-stroke score improved risk prediction within the subgroup of patients classified as moderate risk by the CHA_2_DS_2_-VASc score. The ABC-stroke score also more-accurately identified patients at a low risk of stroke. Given the higher precision of the ABC-stroke score, its use in this important acute cardiac setting may be improve treatment decisions (2, 28).

The dynamic nature of the cardiac biomarkers included in the ABC-stroke score may also be advantageous because changes in these biomarkers may reflect differences in individual stroke risk that may be modified by various factors (29, 30). The latter could be of particular importance since continuous re-evaluation of both stroke and bleeding risk are key for assessing the risks and benefits of anticoagulant therapy (2). However, it should be noted that the benefit-risk ratio of oral anticoagulation in patients labeled moderate risk by the CHA_2_DS_2_-VASc has not been well studied. In some individuals, the anticoagulation risks may outweigh the benefits. Therefore, in acute AF, the decision for long-term anticoagulation must carefully and simultaneously balance individual stroke and bleeding risks. Dynamic bleeding risk assessment could include biomarker-based tools, such tools as the ABC-bleeding score (31).

The stroke incidence rate was 1.66 per 100 person-years in this study. This was lower than expected, especially compared to elderly patients with AF. It is therefore possible that the improved stroke prediction provided by the ABC-stroke score could have an even greater impact in elderly populations, who have a substantially higher risk of adverse events. Despite this lower incidence, our study clearly demonstrated that the ABC-stroke score successfully predicts stroke risk in an emergency department, particularly in cases where the CHA_2_DS_2_-VASc score provides weak anticoagulation guidance. The ABC-stroke score may therefore refine risk stratification. Another issue encountered in routine clinical care is the turnaround time for biomarker tests. However, these biomarkers are usually routinely available in emergency departments with turnaround times of 1–1.5 h and around-the-clock availability. The online availability of the ABC-stroke score nomogram and its calculator also facilitates its clinical application (https://www.ucr.uu.se/en/services/abc-risk-calculators) (6, 8).

### Strengths and Limitations

Clear strengths of the present ABC-stroke score validation are its real-world emergency cohort of patients with AF, the long combined observation period of 3,686 years, several clinically relevant subgroups, and sensitivity analyses that included the ABC-stroke score recalibration based on a recent study of patients with AF not treated with oral anticoagulation therapy (27). In addition, this evaluation utilized the principles and methods for validating and reporting clinical prediction models described by Steyerberg et al. and the TRIPOD consensus statement (17, 18). However, a significant limitation of this study was the lack of information regarding the cause of death in deceased patients.

Finally, it should be noted that the study does not allow conclusions to be drawn regarding patients with classic valvular AF. These patients are only very sparsely represented in our AF registry and therefore could not be investigated separately in the present study.

## Conclusion

In a large series of patients with acute AF treated in the emergency department, the ABC-stroke score provided superior stroke prediction over the CHA_2_DS_2_-VASc score. It also improved the risk stratification of patients labeled moderate risk by the CHA_2_DS_2_-VASc score, thereby easing anticoagulation treatment decision-making.

## Data Availability Statement

The original contributions presented in the study are included in the article/Supplementary Material, further inquiries can be directed to the corresponding author.

## Ethics Statement

The studies involving human participants were reviewed and approved by Ethics Committee of the Medical University of Vienna (1568/2014). The patients/participants provided their written informed consent to participate in this study.

## Author Contributions

JN, MS, and ZH developed the study idea and wrote the final version of the manuscript in critical review. JO, SSc, FC, NB, A-MW, RL, NS, MB, GR, SG, ML, and SSR carried out the main part of the study and helped in writing the manuscript as well as in creating the figures, tables, and graphical and video abstract. HH carried out the statistical tests and analyses and critically revised the final manuscript. MW, HD, and AL participated in the project as senior consultants and critically revised the final manuscript. All authors contributed to the article and approved the submitted version.

## Conflict of Interest

ZH reports personal fees from Boehringer Ingelheim, Bristol-Myers Squibb, Pfizer and Roche Diagnostics for lectures, personal fees from Boehringer Ingelheim, Bristol-Myers Squibb, Pfizer, and Roche Diagnostics for consulting, and grants from the Swedish Society for Medical Research (S17-0133) and the Swedish Heart-Lung Foundation (20170718), outside the submitted work. The remaining authors declare that the research was conducted in the absence of any commercial or financial relationships that could be construed as a potential conflict of interest.

## Publisher's Note

All claims expressed in this article are solely those of the authors and do not necessarily represent those of their affiliated organizations, or those of the publisher, the editors and the reviewers. Any product that may be evaluated in this article, or claim that may be made by its manufacturer, is not guaranteed or endorsed by the publisher.
