# Supplementary figures and images for: The ABC-Stroke Score Refines Stroke Risk Stratification in Patients With Atrial Fibrillation at the Emergency Department
Source: Front Med (Lausanne). 2022 Jun 27;9:830580. doi: 10.3389/fmed.2022.830580 (PMC9271836; doi:10.3389/fmed.2022.830580)

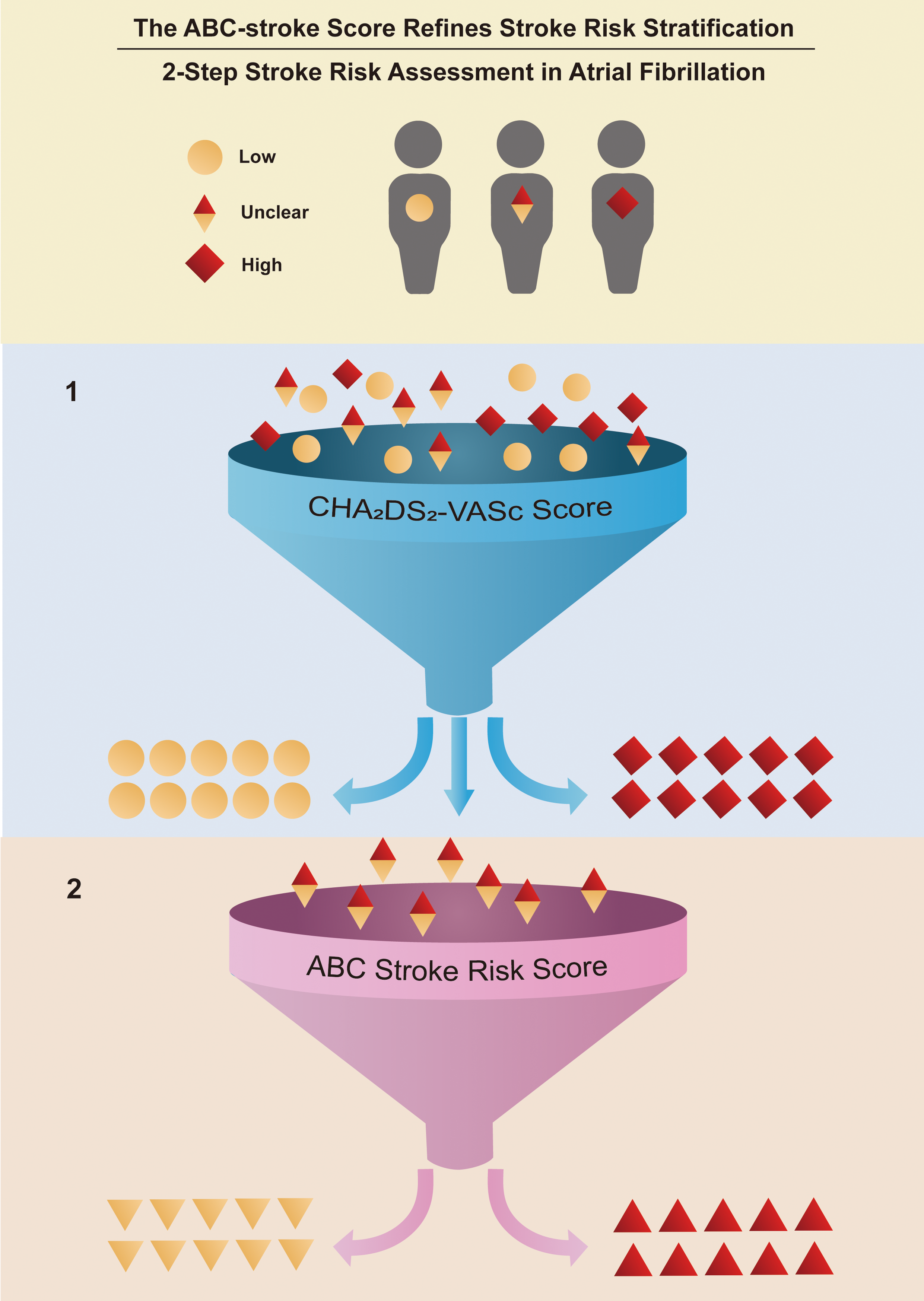

Supplement: Supplementary file 1 [file Image_1.TIF]
