# Supplementary material for: The ABC-Stroke Score Refines Stroke Risk Stratification in Patients With Atrial Fibrillation at the Emergency Department
Source: Front Med (Lausanne). 2022 Jun 27;9:830580. doi: 10.3389/fmed.2022.830580 (PMC9271836; doi:10.3389/fmed.2022.830580)
Supplement: Supplementary file 2 [file Data_Sheet_1.PDF]

The ABC-stroke score refines stroke risk stratification in patients with atrial fibrillation at the emergency department

Niederdöckl et al.

Supplementary Material

Supplementary Table S1 Expanded demographics and baseline characteristics
Supplementary Table S2 Sensitivity analyses for the ABC-stroke score
Supplementary Figure S1 Kaplan-Meier event rates according to CHA2DS2-VASc risk classes

Supplementary Table S1 Expanded demographics and baseline characteristics

Supplemental Extended Table 1	Total		TREATMENT RECOMMENDATION BASED ON CHA2DS2-VASc					
			**No**		**To be considerec**		**Yes**	
					non-sex CHA2DS2-VASc			
			<1		*1*		*>1*	
	n=2108	*available* (n)*	n=219	*available* (n)*	n=437	*available* (n)*	n=1452	*available* (n)*
**General characteristics**								
Age, years (IQR)	68 (59 - 76)		51 (37 - 58)		62 (53 - 68)		73 (66 - 80)	
Female sex, n (%)	911 (43)		87 (40)		163 (37)		661 (46)	
**Comorbidities**								
Heart failure, n (%)	369 (17,5)		4 (1,8)		59 (13,5)		306 (21,1)	
Hypertension, n (%)	1236 (58,6)		0 (0,0)		200 (45,8)		1036 (71,3)	
Diabetes mellitus, n (%)	344 (16,3)		0 (0,0)		12 (2,7)		332 (22,9)	
Prior stroke, n (%)	123 (5,8)		0 (0,0)		0 (0,0)		123 (8,5)	
Coronary artery disease, n (%)	383 (18,2)		0 (0,0)		26 (5,9)		357 (24,6)	
Prior myocardial infarction, n (%)	195 (9,3)		0 (0,0)		7 (1,6)		188 (12,9)	
Peripheral artery disease, n (%)	93 (4,4)		0 (0,0)		3 (0,7)		90 (6,2)	
COPD, n (%)	195 (9,3)		9 (4,1)		17 (3,9)		169 (11,6)	
Valvular disease, n (%)	399 (18,9)		23 (10,5)		80 (18,3)		296 (20,4)	
Current smoker, n (%)	212 (10,1)		36 (16,4)		37 (8,5)		139 (9,6)	
**AF history**								
First AF episode, n (%)	29 (1,4)		1 (0,0)		8 (0,4)		20 (0,9)	
Heart rate, bpm (IQR)	131 (110 - 150)	*1097*	135 (108 - 159)	*148*	134 (119 - 151)	*208*	129 (108 - 147)	*714*
Duration of AF symptoms, h (IQR)	6 (2 - 20)	*846*	4 (1 - 10)	*121*	6 (2 - 18)	*190*	7 (3 - 24)	*535*
**Laboratory**								
Haematocrit, % (IQR)	41 (37 - 45)	*1994*	43 (41 - 46)	*201*	42 (39 - 46)	*411*	40 (36 - 44)	*1382*
WBC, G/l (IQR)	9 (7 - 11)	*2018*	8 (7 - 11)	*202*	8 (7 - 10)	*418*	9 (7 - 11)	*1398*
Creatinine, mg/dl (IQR)	1,0 (0,9 - 1,2)	*2023*	0,9 (0,8 - 1,0)	*203*	1,0 (0,8 - 1,2)	*419*	1,0 (0,9 - 1,3)	*1401*
NT-proBNP, pg/ml (IQR)	636 (150 - 2153)	*2108*	244 (64 - 782)	*219*	465 (133 - 1588)	*437*	932 (192 - 2433)	*1452*
hs-Troponin T, ng/l (IQR)	12 (5 - 23)	*2108*	7 (3 - 13)	*219*	8 (4 - 16)	*437*	13 (6 - 28)	*1452*
CRP, mg/dl (IQR)	0,4 (0,2 - 1,5)	*1969*	0,2 (0,1 - 0,6)	*190*	0,4 (0,1 - 1,2)	*404*	0,5 (0,2 - 1,7)	*1375*
INR, (IQR)	1,4 (1,1 - 2,7)	*965*	1,0 (1,0 - 1,6)	*71*	1,3 (1,0 - 2,5)	*172*	1,6 (1,1 - 2,8)	*722*
**Stroke risk scores**								
CHA2DS2-VASc (IQR)	3 (1 - 4)	*1984*	0 (0 - 0)	*219*	1 (1 - 2)	*437*	3 (3 - 4)	*1328*
ABC-stroke risk, 1-year (IQR)	1 (1 - 1)	*2018*	0 (0 - 1)	*219*	1 (0 - 1)	*437*	1 (1 - 2)	*1452*
**Treatment at admission**								
Anticoagulation, n (%)	673 (31,9)		28 (12,8)		102 (23,3)		543 (37,4)	
Antiplatelet therapy, n (%)	521 (24,7)		54 (24,7)		109 (24,9)		358 (24,7)	
Anticoagulation & antiplatelet therapy, n (%)	60 (2,8)		2 (0,9)		12 (2,7)		46 (3,2)	
**Treatment at discharge**								
Anticoagualtion, n (%)	1107 (52,5)		80 (36,5)		225 (51,5)		802 (55,2)	
Antiplatelet therapy, n (%)	311 (14,8)		11 (5,0)		51 (11,7)		249 (17,1)	
Anticoagulation & antiplatelet therapy, n (%)	237 (11,2)		7 (3,2)		37 (8,5)		193 (13,3)	

Supplementary Table S2 Sensitivity analyses for the ABC-stroke score

Table - C-indices	N	Events	Harrell´s C
**ABC-stroke score**			
All patients with acute AF	2108	61	0.64 (0.57-0.70)
Consider anticoagulation*	437	14	0.66 (0.51-0.80)
**CHA2DS2VASc score**			
All patients with acute AF	2108	61	0.55 (0.49-0.60)
**Sensitivity analyses**			
**Primary Model**			
ABC-stroke score			
Anticoagulation at discharge	1344	39	0.63 (0.56-0.70)
No anticoagulation at discharge	764	22	0.63 (0.52-0.74)
Previous stroke	123	9	0.63 (0.47-0.79)
NSTE-ACS	71	4	0.55 (0.50-0.60)
No NSTE-ACS	2037	57	0.63 (0.57-0.70)
Acute heart failure	117	3	0.70 (0.59-0.81)
**Secondary Model**			
ABC-stroke score			
Recalibrated ABC-stroke score for AF patients without oral anticoagulation treatment**	311	8	0.62 (0.46-0.78)

** non-sex CHA2DS2-VASc = 1*

*** Benz, Hijazi et al. (Circulation, in press)*

Supplementary Figure S1 Kaplan-Meier event rates according to CHA2DS2-VASc risk classes

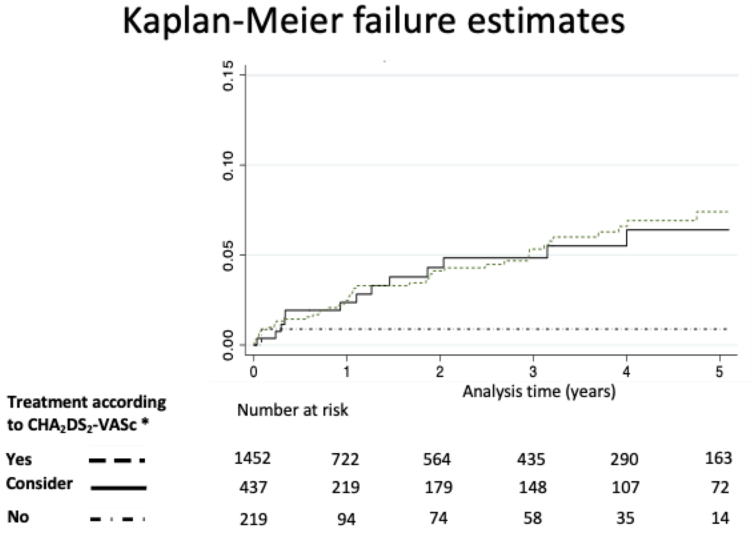

*non-sex CHA_2DS_2-VASc score-based treatment recommendations:
yes = CHA_2DS_2-VASc ≥2
consider= CHA_2DS_2-VASc =1
no = CHA_2DS_2-VASc <1
